# Could effective iodine-131 half-life be extended by lithium carbonate in Graves’ disease patients: Results from a retrospective analysis

**DOI:** 10.17305/bb.2024.10659

**Published:** 2024-12-01

**Authors:** Xuemei Gao, Binbin Wu, Qian Zhou, Yan Liu, Ruihua Wang

**Affiliations:** 1 Department of Nuclear Medicine, The First Affiliated Hospital of Zhengzhou University, Zhengzhou, China; 2 Radiology Department, Luoyang Orthopedic-Traumatological Hospital of Henan Province (Henan Provincial Orthopedic Hospital), Luoyang, China; 3 Medical Imaging Department, Henan University People’s Hospital and Henan Provincial People’s Hospital, Zhengzhou, China

**Keywords:** Effective 131-I half-life, lithium carbonate, Graves’ disease (GD), radioactive iodine therapy (RIT), hyperthyroidism

## Abstract

The effective iodine-131 (I-131) half-life (EHL) plays an important role in the evaluation of radioactive iodine therapy (RIT) for Graves’ disease (GD) patients. It has been observed that the EHL of GD patients varies after taking lithium carbonate. The purpose of this study is to investigate whether EHL can be extended and to identify the predictive factors associated with this outcome. The clinical data of 225 GD patients were retrospectively reviewed. Patients were divided into two groups based on whether the ΔEHL was ≥ 0.5 days. EHL tested after lithium carbonate was defined as Li-EHL. In the univariate analysis, age, sex, thyrotropin receptor antibody (TRAb), thyroglobulin antibody (TgAb), thyroid peroxidase antibody (TPOAb), and baseline-EHL exhibited significant differences between the two groups (*P* < 0.05). Cutoff values of age and baseline-EHL to predict significant EHL extension were 40.5 years and 4.85 days, respectively, as determined by receiver operating characteristic (ROC) curve analysis. Multiple linear regression analysis further revealed that the regression equation, which included age, sex, baseline-EHL, and the FT3, free triiodothyronine (FT4)/free thyroxine(FT3) ratio, was statistically significant (*P* < 0.05). Li-EHL positively correlated with baseline-EHL and the FT4/FT3 ratio, but negatively correlated with age. Li-EHL was also increased in female individuals. In conclusion, age, sex, baseline-EHL, and the FT4/FT3 ratio were associated with Li-EHL in GD patients.

## Introduction

Graves’ disease (GD) is a common autoimmune disorder that causes an overactive thyroid gland, resulting in symptoms, such as weight loss, rapid heartbeat, and bulging eyes. Treatment options include anti-thyroid drugs (ATDs) to regulate thyroid function, radioactive iodine therapy (RIT) to reduce thyroid activity, and surgery to remove part or all of the thyroid gland [1–3]. RIT is a widely used treatment for GD, as it selectively targets and destroys overactive thyroid cells. As a consequence, RIT is often recommended for GD treatment due to its simplicity, higher effectiveness (compared to drug therapy), and fewer side effects [4, 5]. RIT is the first-line treatment option for adults with hyperthyroidism and an alternative treatment for patients with poor ATD therapy response [3, 6–10].

However, there are challenges and difficulties associated with this treatment approach. One of the main challenges is the uptake and effective half-life (EHL) of iodine-131 (I-131) in the thyroid, which has been proven to be an important influencing factor on RIT efficacy [11]. The physical half-life of radio iodine is approximately 8.1 days. Several factors can shorten the EHL, including an individual’s metabolic rate, thyroid function, and age. Before implementing RIT, the following measures are routinely taken to increase the uptake of I-131 and thus extend EHL in the body, including a low iodine diet and avoiding certain drugs (e.g., methimazole, propylthiouracil, and iodine-containing contrast agents) [11]. Lithium carbonate, a medication commonly used to treat bipolar disorder, has also been shown to be effective in the treatment of hyperthyroidism, specifically GD [12, 13]. It also stimulates bone marrow hematopoietic function, promotes the proliferation of blood cells, and reduces the incidence of bleeding. In addition, lithium carbonate could treat hyperthyroidism by inhibiting the release of thyroid hormones [14]. Currently, lithium carbonate is used for short-term hyperthyroidism management due to its ability to increase thyroid I-131 retention and reduce the treatment dosage of I-131 [6]. Lithium carbonate has also been used to increase the EHL of I-131 in thyroid treatment [15]. According to reports, the dosage of lithium carbonate used as an adjuvant in the treatment of hyperthyroidism ranges from 600 mg/day to 900 mg/day, and the duration of use ranges from 6 days to 3 weeks [16]. Side effects of short-term lithium therapy were virtually absent. Lithium carbonate enhances the effectiveness of radioiodine therapy, in terms of prompter control of hyperthyroidism, in patients with small or large goiters [17].

Due to the low toxicity and safety of the short-term application of lithium carbonate, its combination with I-131 in the treatment of hyperthyroidism is increasingly attracting clinical attention [11, 18, 19]. However, some patients who receive lithium carbonate pretreatment before I-131 therapy do not exhibit longer I-131 retention in clinical application. Therefore, additional research is needed to clarify the characteristics of hyperthyroidism patients who can benefit from lithium carbonate adjunct I-131 for effective therapy. To clarify the above issues, we performed a retrospective study involving 225 patients to elucidate the effects and possible risk factors of lithium carbonate pretreatment on the EHL of GD patients.

## Materials and methods

### Study participants

This study was conducted from February 2015 to September 2021 and included 225 GD patients (76 males and 149 females) who met the inclusion criteria were included. The inclusion criteria were: (1) a definite diagnosis of GD and (2) compliance with medical advice regarding lithium carbonate. Patients were excluded for any of the following conditions: (1) thyrotoxicosis caused by Plummer’s disease, toxic multinodular goiter, or thyroiditis; (2) severe liver or kidney dysfunction, acute myocardial infarction, heart failure, or other severe diseases; (3) serious adverse digestive or nervous system reactions to lithium carbonate; and (4) pregnancy or lactation. All enrolled patients stopped taking ATDs, other drugs, or foods that might affect thyroid I-131 uptake.

### Baseline patient characteristics

The baseline characteristics collected included name, sex, age, duration of hyperthyroidism, and treatment history of ATD or RIT. Serum free triiodothyronine (FT3), free thyroxine (FT4), and thyrotropin (TSH) levels were determined using a chemiluminescence method according to the manufacturer’s protocol (by UniCel DxI 800 Access, Beckman, USA). The reference ranges for FT3, FT4, and TSH are 3.28–6.47 pmol/L, 7.9–18.4 pmol/L, and 0.56–5.91 µIU/mL, respectively. Thyroid peroxidase antibody (TPOAb), thyroglobulin antibody (TgAb), and thyrotropin receptor antibody (TRAb) levels were determined using an electrochemiluminescence immunoassay according to the manufacturer’s protocol (by Cobas 801, Roche, Germany), with reference ranges of 0–34 IU/mL, 3.7–77 µg/L, and 0–1.75 IU/L, respectively. Routine blood tests, liver and kidney function tests, and electrocardiograms (ECG) were routinely performed to evaluate patients’ physical condition.

### Thyroid volume/weight

The thyroid volume was measured using three different methods: ^99m^Tc-thyroid scintigraphy, thyroid ultrasound, and palpation. Thyroid scintigraphy was carried out to observe the distribution of radioactivity, determine the functional state of nodules, and exclude acute or subacute thyroiditis. Image acquisition was performed using SPECT/CT (NM/CT 670, GE Healthcare) 30–60 min after the injection of 185 MBq ^99m^Tc-NaTcO_4_ (provided by Atomic High Tech, Beijing). Subsequently, the acquired images were processed and analyzed by two senior nuclear medicine attending physicians using the Thyroid software at Xeleris Functional Imaging Workstation (Xeleris Version 4.0, GE Healthcare), and the estimated weight was obtained from the ^99m^Tc-thyroid scintigraphy. Thyroid ultrasonography was performed using a 7–14 MHz probe (Toshiba, Aplio500, Japan). The length, width, and thickness of the two lobes of the thyroid gland were measured. Palpation was performed by two experienced physicians. The average weight of the thyroid gland was assessed according to the methods mentioned above.

### Calculation of radioactive iodine uptake and EHL of I-131

Radioactive iodine uptake (RAIU) was measured at 2, 4, and 24 h after the administration of a tracer I-131 dose (74–370 kBq) using the thyroid-uptake system (Zhongke Zhongjia, MN-6110B, China). The normal reference values of RAIU at 2, 4, and 24 h are 7%–15%, 12%–25%, and 20%–38%, respectively. As previously reported, the negative correlation between the EHL and the conversion rate can be expressed by the regression equation *y* ═ –4.5551*x* + 0.1693, where *x* represents the conversion rate [20]. The EHL of *I* ═ 131 in the thyroid was calculated as mentioned above.

### Lithium carbonate pretreatment

Data were collected at baseline and after the completion of a 7-day administration of lithium carbonate. Patients were treated with 250 mg of lithium carbonate (provided by Hunan Qianjin Xiangjiang Pharmaceutical Co., Ltd.) orally three times a day for a week. During this period, adverse drug reactions caused by lithium carbonate were also recorded. Lithium levels in the blood were not routinely monitored, except if adverse effects occurred. In fact, none of the patients complained about adverse reactions after lithium carbonate treatment and no patient discontinued lithium carbonate treatment due to adverse reactions. On the 8th day, patients’ data, including FT3, FT4, TSH, RAIUs, and EHL were re-evaluated. The EHL tested after seven days of lithium carbonate was defined as Li-EHL. ΔEHL was defined as the difference between Li-EHL and baseline-EHL.

### Quantification of each clinical data and laboratory tests

Independent variables were: X1 ═ age; X2 ═ sex: X2 ═ 0, if sex is male, and X2 ═ 1, if sex is female; X3 ═ estimated thyroid weight (g); X4 ═ 0, if TRAb is negative, and X4 ═ 1, if TRAb is positive; X5 ═ TPOAb; X6 ═ baseline-EHL; X7 ═ FT4/FT3 ratio.

Dependent variable: Y ═ Li-EHL.

### Ethical statement

Approval for this retrospective observational study was obtained from the Ethics Committee of the First Affiliated Hospital of Zhengzhou University (ethics committee approval code: 2024-KY-0016, Date: March 28, 2024) in accordance with the Declaration of Helsinki.

**Table 1:** Baseline characteristics of patients

**Variables**	**Overall (*n* ═ 225)**	**ΔEHL≥0.5 d (*n* ═ 90)**	**ΔEHL<0.5 d (*n* ═ 135)**	**t/χ^2^**	***P***
Age (years)	41.26 ± 12.03	30.41 ± 6.55	48.49 ± 9.04	17.385	0.001^*^
*Sex*					
Male	76 (33.78%)	18 (8.00%)	58 (25.78%)	12.730	0.001^*^
Female	149 (66.22%)	72 (32.00%)	77 (34.22%)		
*TRAb*					
Positive	217 (96.44%)	90 (40.00%)	127 (56.44%)	5.530	0.019^*^
Negative	8 (3.56%)	0 (0.00%)	8 (3.56%)		
*TgAb*					
Positive	159 (70.22%)	72 (32.00%)	87 (38.67%)	6.304	0.012^*^
Negative	66 (29.33%)	18 (8.00%)	48 (21.33%)		
TPOAb (IU/mL)	288.72 ± 207.690	357.84 ± 212.100	242.63 ± 192.038	4.144	0.001^*^
Baseline-EHL (days)	5.02 ± 1.108	4.86 ± 1.331	5.12 ± 0.921	10.582	0.001^*^

Continuous variables are expressed as mean ± SD or count (%). ^*^P < 0.05. TRAb: Thyrotropin receptor antibody; TgAb: Thyroglobulin antibody; TPOAb: Thyroid peroxidase antibody; EHL: Effective half-life; d: Days.

### Statistical analysis

Data were analyzed using the SPSS software, V13.0 (SPSS Inc, Chicago, IL, USA). Continuous normally distributed data were expressed as mean ± standard deviation (SD). Changes in serum FT3 and FT4 levels before and after lithium carbonate administration were analyzed using the paired *t*-test. Differences in the EHL levels between groups were analyzed using the *t*-test and chi-square test. Potential factors predicting the Li-EHL were evaluated using multiple linear regression analyses. Receiver operating characteristic (ROC) curves were carried out to determine cut-off values for selected variables. *P <* 0.05 was considered statistically significant.

## Results

### Baseline patient characteristics and comparisons between groups

A total of 225 individuals conformed to the inclusion criteria with a mean age of 41.26 ± 12.03 years (ranging from 12 to 68 years). There were 76 males (range 12–66 years, 41.21 ± 12.22 years) and 149 females (range 16–68 years, 41.28 ± 11.97 years), with a male-to-female ratio of 1:1.96. The duration of hyperthyroidism varied from 1 month to 242 months, with an average of 39.59 ± 46.10 months. One hundred and fifty-six patients (69.33%) had a history of ATD treatment, while 69 (30.67%) did not. Twenty-eight (12.44%) patients had previously received RIT, while 197 (87.56%) had not.

The EHL reported in this article is in days, abbreviated as “d.” All patients were divided into two groups based on the changes in EHL levels after lithium carbonate treatment: ΔEHL ≥ 0.5 d group and ΔEHL < 0.5 d group. The ΔEHL was 1.40 ± 0.726 d (range 0.50–3.65 d) in the former group, and the ΔEHL was −0.24 ± 0.517 d (range −1.75 to −0.50 d) in the latter group. There was no statistically significant difference in thyroid weight, FT3, FT4, TSH, TRAb, TgAb levels, duration of hyperthyroidism, RAIU tested at 2h, 4h, and24h (*t* ═ 0.059–1.740), and history of ATD/RIT treatment (χ^2^ ═ 0.906–1.740) between the two groups (all *P* > 0.05). The baseline characteristics of patients and univariate analysis are summarized in Table 1.

### Changes of free thyroid hormone levels, RAIUs, and EHL in patients after lithium carbonate treatment

FT3, FT4, TSH, and RAIU at 2, 4, and 24 h were remeasured after a 7-day lithium carbonate treatment, and Li-EHL was also determined. We found that serum FT3 andFT4 levels were significantly reduced after lithium carbonate treatment (*t* ═ 2.864–3.663, all *P* < 0.05). The levels of FT3 and FT4 decreased by 20.85% and 11.93%, respectively. RAIU measured at 24 h increased by 9.93% (*t* ═ 5.681, *P* < 0.05), while no significant changes in RAIUs measured at 2 and 4 h were observed (*t* ═ 0.289∼1.347, *P* > 0.05). Li-EHL increased by 8.17% after lithium treatment (*t* ═ 6.148, *P* < 0.05). Detailed data are presented in Table 2.

**Table 2:** Comparison of clinical data before and after lithium carbonate treatment in patients

**Variable**	**Before lithium**	**After lithium**	***t***	***P***
FT3 (pmol/L)	24.03 ± 11.89	19.17 ± 8.82	3.663	0.002^*^
FT4 (pmol/L)	52.61 ± 16.67	47.33 ± 15.66	2.864	0.005^*^
TSH (mU/L)	0.02 ± 0.02	0.02 ± 0.02	1.254	0.176
2h RAIU (%)	52.25 ± 24.01	51.63 ± 19.03	0.289	0.773
4h RAIU (%)	66.73 ± 27.25	68.66 ± 23.66	1.347	0.179
24h RAIU (%)	71.17 ± 21.47	78.24 ± 14.81	5.681	0.001^*^
EHL (days)	5.02 ± 1.108	5.43 ± 0.835	6.148	0.001^*^

^*^P < 0.05. FT3: Free triiodothyronine; FT4: Free thyroxine; TSH: Thyroid-stimulating hormone; RAIU: Radioactivity iodine uptake; EHL: Effective half-life.

### Multifactor analysis of all casual factors

Multiple linear regression analysis was carried out to screen all independent variables, and results suggested that the factors that have linear correlation with Li-EHL include: X1 (age), X2 (sex), X6 (baseline-EHL), and X7 (FT4/FT3 ratio). The regression equation is:

*Y* ═ 2.643–0.063X1 + 0.477X2 + 0.001X3 + 0.229X4 + 0.015X5 + 0.858X6 + 0.184X7 (Adjusted R^2^ ═ 0.841, *F* ═ 170.089, *P* < 0.001) (Table 3).

**Table 3:** Variables and constants in the logistic resection equation

**Model**	**Variable**	**B**	***P***	**95% CI**
X1	Age	−0.042	0.001*	−0.049 to −0.036
X2	Sex	−0.411	0.001*	−0.595 to −0.228
X3	Weight	0.000	0.564	−0.004 to 0.002
X4	TRAb	−0.301	0.002*	−0.487 to −0.114
X5	TPOAb/100	0.015	0.241	−0.010 to 0.039
X6	Baseline-EHL	0.805	0.001*	0.751 to 0.860
X7	FT4/FT3 ratio	0.180	0.001*	0.121 to 0.240

^*^P < 0.05. TRAb: Thyrotropin receptor antibody; TPOAb: Thyroid peroxidase antibody; EHL: Effective half-life; FT3: Free triiodothyronine; FT4: Free thyroxine.

### Receiver operating characteristics (ROC) curves

We used the two influential factors to draw ROC curves and tested them based on the areas under the curve. It was shown that they were all useful in predicting Li-EHL (Table 4).

**Table 4:** Estimated AUC and cut-off values by ROC curves

**Variable**	**AUC**	**Cut-off**	**Sensitivity**	**Specificity**	**Youden index**	***P***	**95% CI**
Age	0.966	40.5	0.989	0.800	0.789	0.001^*^	0.948 to 0.985
Baseline-EHL	0.854	4.85	0.833	0.726	0.559	0.001^*^	0.804 to 0.904

*P < 0.05. AUC: Area under the curve; CI: Confidence incidence; TPOAb: Thyroid peroxidase antibody; EHL: Effective half-life.

## Discussion

RIT is a first-line treatment strategy for GD, especially in patients with ATD failure, allergies, combined liver injury, leukopenia/deficiency, or disease recurrence. Although RIT is a common and effective treatment for GD, it is not without its challenges and difficulties. Uptake efficiency and EHL in the thyroid are key factors affecting the I-131 efficacy in treating GD. Increasing the dose of I-131 seems to be one of the solutions to address this challenge, which has both benefits and drawbacks. While it improves treatment outcomes and provides faster relief of symptoms, it also carries risks, such as an increased likelihood of hypothyroidism and potential radiation exposure to other organs. Several studies have demonstrated that short-term pretreatment with lithium carbonate could prolong the EHL and result in superior cure rates with lower I-131 doses [21]. Nevertheless, other reports have indicated that lithium carbonate had little effect on the efficacy of I-131 treatment [22]. Owing to the controversial and insufficient study results, the treatment strategy of I-131 pretreatment with lithium carbonate is not widely used in clinical practice [6, 7, 16, 23]. Therefore, more detailed data are needed to elucidate the effectiveness and predictive factors of lithium carbonate pretreatment. In the present study, we found that lithium carbonate pretreatment increased the EHL in 65.33% (147/225) of patients, and 61.22% (90/147) of them had an extended EHL of more than 0.5 days. These data further demonstrate the significance of lithium carbonate as an adjunct for RIT. More importantly, we further analyzed the underlying clinical factors which could predict the Li-EHL after lithium carbonate pretreatment in GD. The results indicated that younger age (<40.5 years), female sex, longer baseline-EHL (>4.85 days), and higher FT4/FT3 ratio were predictive factors for extended Li-EHL. These results underscore the critical role of lithium carbonate for RIT in hyperthyroidism and suggest the significance of these predictive factors in this treatment strategy, simultaneously providing a basis for the rational administration of lithium carbonate, which has not been reported previously.

Previous studies suggested that lithium carbonate could increase iodine retention in the thyroid, but the influence of lithium carbonate on iodine uptake is not well studied. Kumar et al. [24] reported a significant increase in the thyroid counts in lithium-treated rats after I-131 administration at 4 and 24 h when compared to the control group. In the present study, the data suggested that lithium carbonate treatment had little effect on the RAIUs at 2 and 4 h, while RAIU measured at 24 h increased significantly. It is reported that 24-h RAIU is an independent predictor of I-131 treatment efficiency, and high 24-h RAIU is associated with better treatment outcomes. Therefore, lithium carbonate is likely to extend EHL by increasing RAIU at 24 h, while longer EHL has been shown to be associated with higher RIT efficacy [25].

The reason why lithium carbonate has a more significant impact on iodine metabolism in young female patients is likely due to the fact that lithium can interfere with the thyroid gland’s ability to uptake and utilize iodine [26]. This can lead to disruptions in thyroid hormone production and metabolism, particularly in individuals who are already more susceptible to thyroid disorders such as young females [27]. There is limited research in this area, and further studies are needed to confirm these results.

Positivity for TPOAb is common in patients with GD. It is reported that TPOAb is an independent predictor of long-term remission after ATD treatment [28]. Previous studies have also confirmed that prolonged EHL is a predictive factor for the effectiveness of RIT [25]. However, the correlation between TPOAb positivity and the effectiveness of I-131 treatment is still largely unknown. Our present study revealed that the ΔEHL ≥ 0.5 d group had higher titers of TPOAb than the ΔEHL < 0.5 d group (*t* ═ 4.144, *P* < 0.001). Unfortunately, in the multifactor linear regression equation, the *P* value of TPOAb was greater than 0.05 and had no statistical significance. The relationship between lithium carbonate and TPOAb may provide an innovative therapeutic approach for these patients.

As with most retrospective studies, this study has certain limitations. Firstly, the patient’s blood lithium concentration was not routinely monitored in this study, and it remains unknown whether there is a linear correlation between blood lithium concentration and laboratory tests. Secondly, follow-up data after RIT were not included in this study, so the relationship between the use of lithium carbonate and the improvement of disease remission time and remission rate after RIT is still unknown.

## Conclusion

In conclusion, we assessed the impact of lithium carbonate pretreatment on I-131 EHL and analyzed the related predictive factors in patients with GD. Extended Li-EHL is expected in patients presenting with long baseline-EHL, and a high FT4/FT3 ratio, particularly in female GD patients under 40.5 years. These results may provide theoretical support and a useful adjuvant treatment option for patients with hyperthyroidism preparing for RIT. Therefore, further prospective studies with larger samples, multicenter participation, and long-term detailed follow-up data are needed in the future.

## Data Availability

The datasets generated during and/or analyzed during the current study are not publicly available, but are available from the corresponding author on reasonable request.

## References

[1] Lee SY, Pearce EN (2023 Oct). Hyperthyroidism: a review. JAMA.

[2] Conaglen HM, Tamatea JAU, Conaglen JV, Elston MS (2018 Jun). Treatment choice, satisfaction and quality of life in patients with Graves’ disease. Clin Endocrinol (Oxf).

[3] Mm K, Da M, Hj D (1998 Mar). Treatment of hyperthyroidism with radioactive iodine. Endocrinol Metabolism Clin North America.

[4] Kim BW (2022 Jan). Does radioactive iodine therapy for hyperthyroidism cause cancer?. J Clin Endocrinol Metab.

[5] Evron JM, Esfandiari NH, Papaleontiou M (2020 Oct). Cancer incidence and mortality following treatment of hyperthyroidism with radioactive iodine. Curr Opin Endocrinol Diabetes Obes.

[6] Ross DS, Burch HB, Cooper DS, Greenlee MC, Laurberg P, Maia AL (2016 Oct). 2016 American thyroid association guidelines for diagnosis and management of hyperthyroidism and other causes of thyrotoxicosis. Thyroid.

[7] Kahaly GJ, Bartalena L, Hegedüs L, Leenhardt L, Poppe K, Pearce SH (2018 Aug). 2018 European thyroid association guideline for the management of graves’ hyperthyroidism. Eur Thyroid J.

[8] Kaplan MM, Meier DA, Dworkin HJ (1998 Mar). Treatment of hyperthyroidism with radioactive iodine. Endocrinol Metab Clin North Am.

[9] Nelson TS, Raiman RJ, Clark DE (1954 Mar). Radioactive iodine in hyperthyroidism. Med Clin North Am.

[10] Mccullagh EP (1952 Oct). Radioactive iodine in the treatment of hyperthyroidism. Ann Intern Med.

[11] Lazarus JH (2009 Dec). Lithium and thyroid. Best Pract Res Clin Endocrinol Metab.

[12] Kibirige D, Luzinda K, Ssekitoleko R (2013 Feb). Spectrum of lithium induced thyroid abnormalities: a current perspective. Thyroid Res.

[13] Lerena VS, León NS, Sosa S, Deligiannis NG, Danilowicz K, Rizzo LFL (2022). Lithium and endocrine dysfunction [Internet]. Medicina (B Aires).

[14] Turner JG, Brownlie BE, Rogers TG (1976 Mar). Lithium as an adjunct to radioiodine therapy for thyrotoxicosis. Lancet.

[15] Dunkelmann S, Künstner H, Nabavi E, Eberlein U, Groth P, Schümichen C (2006). Lithium as an adjunct to radioiodine therapy in Graves’ disease for prolonging the intrathyroidal effective half-life of radioiodine. Useful or not?. Nuklearmedizin.

[16] Abd-ElGawad M, Abdelmonem M, Ahmed AE, Mohammed OM, Zaazouee MS, Assar A (2021 Apr). Lithium carbonate as add-on therapy to radioiodine in the treatment on hyperthyroidism: a systematic review and meta-analysis. BMC Endocr Disord.

[17] Bogazzi F, Bartalena L, Brogioni S, Scarcello G, Burelli A, Campomori A (1999 Feb). Comparison of radioiodine with radioiodine plus lithium in the treatment of Graves’ hyperthyroidism. J Clin Endocrinol Metab.

[18] Martin NM, Patel M, Nijher GMK, Misra S, Murphy E, Meeran K (2012 Oct). Adjuvant lithium improves the efficacy of radioactive iodine treatment in Graves’ and toxic nodular disease. Clin Endocrinol (Oxf).

[19] Sekulić V, Rajić M, Vlajković M, Ilić S, Stević M, Kojić M (2017 Dec). The effect of short-term treatment with lithium carbonate on the outcome of radioiodine therapy in patients with long-lasting Graves’ hyperthyroidism. Ann Nucl Med.

[20] Tian J, Kang Z (2012 Dec). Iodine-131 treatment of Graves’ hyperthyroidism: new means of effective half-life measurement. China Med Pharmacy.

[21] Chouhan A, Abhyankar A, Basu S (2016 Jan). The feasibility of low-dose oral lithium therapy and its effect on thyroidal radioiodine uptake, retention, and hormonal parameters in various subcategories of hyperthyroid patients: a pilot study. Nucl Med Commun.

[22] Kessler L, Palla J, Baru JS, Onyenwenyi C, George AM, Lucas BP (2014 Jul). Lithium as an adjunct to radioactive iodine for the treatment of hyperthyroidism: a systematic review and meta-analysis. Endocr Pract.

[23] Suwansaksri N, Preechasuk L, Kunavisarut T (2018). Nonthionamide drugs for the treatment of hyperthyroidism: from present to future. Int J Endocrinol.

[24] Kumar SB, Kamal R, Khan A, Chadha VD (2019 May). Dose optimization of lithium to increase the uptake and retention of I-131 in rat thyroid. Radiat Environ Biophys.

[25] Zheng W, Jian T, Guizhi Z, Zhaowei M, Renfei W (2012 Jan). Analysis of ^131^I therapy and correlation factors of Graves’ disease patients: a 4-year retrospective study. Nucl Med Commun.

[26] Czarnywojtek A, Zgorzalewicz-Stachowiak M, Czarnocka B, Sawicka-Gutaj N, Gut P, Krela-Kazmierczak I (2020 Apr). Effect of lithium carbonate on the function of the thyroid gland: mechanism of action and clinical implications. J Physiol Pharmacol.

[27] De Leo S, Lee SY, Braverman LE (2016 Aug). Hyperthyroidism. Lancet.

[28] Gokbulut P, Koc G, Kuskonmaz SM, Onder CE, Omma T, Fırat S (2023). High thyroperoxidase antibody titers may predict response to antithyroid drug treatment in graves disease: a preliminary study. Acta Endocrinol (Buchar).

